# Pretreatment Voiding Volume as a Predictor of Post-brachytherapy Urinary Quality of Life in Patients With Prostate Cancer: A Retrospective Study

**DOI:** 10.7759/cureus.91946

**Published:** 2025-09-09

**Authors:** Naoto Wakita, Jun Teishima, Takuto Hara, Taisuke Tobe, Hideto Ueki, Kotaro Suzuki, Tomoaki Terakawa, Akihisa Yao, Hideaki Miyake

**Affiliations:** 1 Division of Urology, Department of Surgery, Kobe University Graduate School of Medicine, Kobe, JPN

**Keywords:** brachytherapy, prostate cancer, quality of life, urinary function, voiding volume

## Abstract

Introduction

This study was performed to investigate the relationship between changes in urination-related quality of life (QOL) after brachytherapy and preoperative findings, including urinary function, urination scores, and patient background.

Methods

This retrospective study included 193 patients with localized prostate cancer who underwent brachytherapy at our institution. Urination-related QOL was assessed using the urinary domain of the Expanded Prostate Cancer Index Composite (EPIC) score, measured before treatment and 3, 6, and 12 months after treatment. Changes in EPIC scores from baseline were analyzed, and preoperative urination-related parameters - including prostate volume, maximum urinary flow rate, voiding volume, age, International Prostate Symptom Score (IPSS), and Overactive Bladder Symptom Score (OABSS) - were compared with post-treatment QOL outcomes.

Results

The urination-related EPIC score was lowest at three months post-treatment but gradually improved toward baseline levels. Patients with a preoperative voiding volume of <192 mL showed significantly greater declines in EPIC scores at 3, 6, and 12 months post-treatment. Additionally, those with an IPSS of <7 or an OABSS of <3 had significantly greater declines at six months, but these differences were not observed at 12 months. Prostate volume, maximum urinary flow rate, and age did not significantly impact changes in EPIC scores.

Conclusion

Preoperative voiding volume was significantly associated with changes in post-treatment urination-related QOL. These findings suggest that preoperative urination-related parameters may help predict post-treatment QOL, highlighting the importance of evaluating pretreatment urinary function when selecting treatments for patients with localized prostate cancer.

## Introduction

Prostate cancer is one of the most prevalent malignancies among men worldwide, with localized prostate cancer accounting for a significant portion of cases [1]. Among the various treatment options, low-dose-rate (LDR) brachytherapy - an approach that involves implanting permanent radioactive seeds such as I-125 and Pd-103 into the prostate - is widely recognized as an effective treatment [2]. This method is particularly beneficial for patients who are not candidates for surgery because of factors such as advanced age, significant comorbidities, or personal preferences regarding invasiveness.

In cases of low- to intermediate-risk localized prostate cancer, LDR brachytherapy has been shown to provide outcomes and therapeutic efficacy comparable to those of external beam radiation therapy (EBRT) and radical prostatectomy [3]. While the prognosis for patients with localized prostate cancer is generally favorable regardless of the treatment modality, quality of life (QOL) after treatment has become a critical factor in decision-making, as it significantly influences the patient’s post-treatment experience.

Different treatments have varying effects on urinary symptoms - such as incontinence, frequency, and urgency - as well as on sexual and bowel functions, making the prediction of post-treatment QOL an essential aspect of pretreatment planning [4]. Research has shown a correlation between pretreatment lower urinary tract symptoms and post-LDR brachytherapy QOL, particularly when assessed using subjective scoring systems such as the International Prostate Symptom Score (IPSS) and the Overactive Bladder Symptom Score (OABSS) [5,6]. However, studies examining the relationship between objective measurements and changes in QOL after treatment remain limited.

Therefore, in this study, we evaluated urinary-related QOL in patients undergoing LDR brachytherapy and investigated the impact of preoperative urination-related parameters - such as prostate volume, maximum urinary flow rate, voiding volume, age, IPSS, and OABSS - on post-treatment QOL. By identifying predictive factors for urinary function, we seek to improve the accuracy of post-treatment QOL predictions, ultimately supporting personalized treatment planning for patients with localized prostate cancer.

## Materials and methods

Study design and setting

This retrospective study was conducted at a single institution in Japan. Approval was obtained from the Kobe University Clinical Research Ethical Committee (approval number B240033) before the study’s initiation.

Patients with localized prostate cancer who underwent LDR brachytherapy at Kobe University Hospital, Kobe, Japan, between February 2009 and May 2022 were included in this study. Patients were excluded if they did not have at least one year of follow-up after treatment, or if their pretreatment and post-treatment QOL scores could not be assessed. All included patients had histologically confirmed prostate adenocarcinoma before initiating treatment. Localized prostate cancer was defined as the absence of local progression on magnetic resonance imaging and no metastasis on computed tomography scans or bone scintigraphy.

All patients underwent uroflowmetry just before seed implantation, with assessments of voiding volume and maximum urinary flow rate. Prostate volume was measured during the pre-planning process to determine the number of implanted seeds. For patients receiving brachytherapy alone, a prescribed dose of 145 Gy was delivered. When brachytherapy was combined with EBRT, the brachytherapy component delivered 110 Gy, supplemented by 45 Gy in 25 fractions from EBRT. For patients with prostate volumes exceeding 30 mL, luteinizing hormone-releasing hormone agonists were administered for three to six months to reduce prostate size before initiating treatment. According to the National Comprehensive Cancer Network guidelines [7], low-risk cases were treated with brachytherapy alone. For intermediate- and high-risk cases, treatment decisions - including the combination of brachytherapy, EBRT, and hormonal therapy - were made at the physician’s discretion. After treatment, alpha-1 blockers were prescribed to manage urinary symptoms until improvement was observed. Prostate-specific antigen levels were regularly monitored. In cases of recurrence, systemic therapies, including hormonal therapy, were considered.

Patient characteristics

We retrospectively collected the following patient characteristics from the medical records: age, Eastern Cooperative Oncology Group performance status, body mass index, urinary flow measurements (voiding volume and maximum urinary flow rate), pretreatment IPSS, and pretreatment OABSS. Additional data included the administration of luteinizing hormone-releasing hormone agonists, pretreatment prostate volume, and the use of EBRT.

Tumor characteristics, including tumor stage at diagnosis, Gleason grade, and National Comprehensive Cancer Network risk classification, were recorded. Blood tests were conducted on all patients before treatment to assess prostate-specific antigen levels, which were then regularly monitored post-treatment to detect recurrence.

QOL score

QOL was assessed before treatment and at 3, 6, and 12 months after completing radiation therapy using the Expanded Prostate Cancer Index Composite (EPIC). This assessment evaluates four domains: urinary (seven items), bowel (nine items), sexual (nine items), and hormonal (six items). Each domain score ranges from 0 (worst) to 100 (best) [8,9].

Statistical analysis

Continuous variables are reported as mean ± standard deviation for normally distributed data, and as median and interquartile range for non-normally distributed data. The Wilcoxon signed-rank test was used to compare continuous variables within paired groups, while the Mann-Whitney U test and Kruskal-Wallis test were used to evaluate differences between independent groups. Categorical variables were analyzed using the chi-square test. Cut-off values for each parameter were determined using receiver operating characteristic (ROC) curve analysis, with the recovery of EPIC scores to baseline levels at 12 months after treatment as the endpoint. A p-value of < 0.05 was considered statistically significant. All statistical analyses were performed using EZR [10], a graphical user interface for R designed to incorporate statistical functions commonly used in biostatistics.

## Results

In total, 193 patients with localized prostate cancer who underwent brachytherapy were included in this study. The patients’ characteristics are summarized in Table 1. The median follow-up duration was 62.3 months. No cases of cancer recurrence were observed within the first 12 months after treatment, and no cancer-related deaths occurred during the study period.

**Table 1:** Patient characteristics ADT, Androgen Deprivation Therapy; BMI, Body Mass Index; ECOG-PS, Eastern Cooperative Oncology Group Performance Status; EBRT, External Beam Radiation Therapy; IQR, Interquartile Range; IPSS, International Prostate Symptom Score; OABSS, Overactive Bladder Symptom Score

Characteristics	Value
No. of patients	193
Age (yr), median (IQR)	69 (64-73)
ECOG-PS, median (IQR)	1 (0-1)
BMI, median (IQR)	23.4 (21.5-25.2)
NCCN risk classification, n (%)	
Low	91 (47.2)
Intermediate	96 (49.7)
High	6 (3.1)
Prostate volume (mL), median (IQR)	27 (23-32)
Neoadjuvant ADT, n (%)	79 (40.9)
EBRT combination, n (%)	13 (6.8)
Baseline IPSS, median (IQR)	7 (4-12)
Baseline OABSS, median (IQR)	3 (2-4)
Maximum urinary flow rate (mL/s), median (IQR)	15.2 (9.6-20.5)
Voiding volume (mL), median (IQR)	208 (115-296)

**Figure 1:**
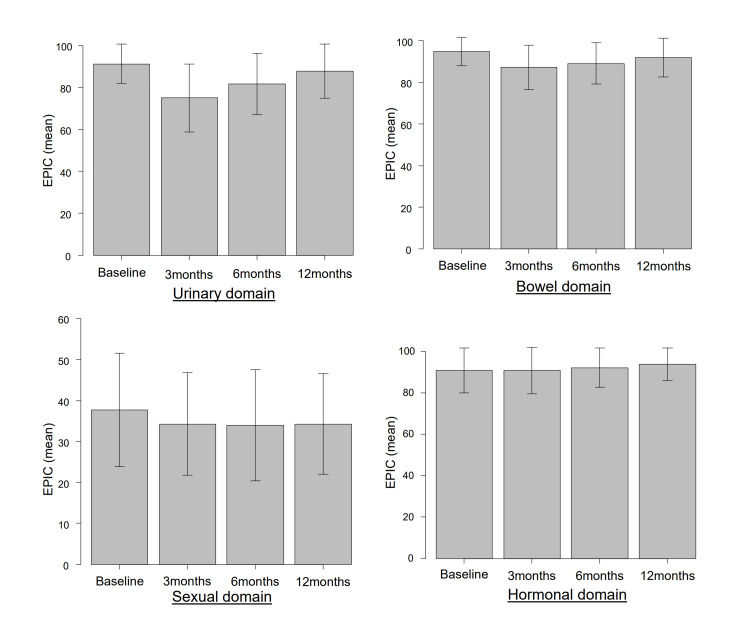
Trends in mean the EPIC scores for the urinary, bowel, sexual, and hormonal domains EPIC, Expanded Prostate Cancer Index Composite

Table 2 and Figure 1 present the mean EPIC scores for the urinary, bowel, sexual, and hormonal domains before treatment and at 3, 6, and 12 months after treatment. In the urinary and bowel domains, the EPIC scores were lowest at three months post-treatment but gradually improved over time, approaching baseline levels. However, at 12 months post-treatment, the scores remained significantly lower than baseline. For the sexual domain, the EPIC scores showed a significant decline from baseline at three months post-treatment and did not improve through 12 months post-treatment. By contrast, in the hormonal domain, the EPIC scores at three and six months post-treatment did not differ significantly from baseline. However, by 12 months post-treatment, a significant improvement from baseline was observed.

**Table 2:** The EPIC scores for the urinary, bowel, sexual, and hormonal domains * indicates statistical significance with p < 0.05. BL, Baseline; EPIC, Expanded Prostate Cancer Index Composite; SD, Standard Deviation

The EPIC scores							
Domains	Baseline (Mean ± SD)	3 months (Mean ± SD)	p-value (vs BL)	6 months (mean ± SD)	p-value (vs BL)	12 months (Mean ± SD)	p-value (vs BL)
Urinary domain	91.3 ± 9.4	75.1 ± 16.2	<0.01*	81.6 ± 14.6	<0.01*	87.8 ± 12.9	<0.01*
Bowel domain	94.8 ± 6.7	87.2 ± 10.7	<0.01*	89.0 ± 9.9	<0.01*	92.0 ± 9.3	<0.01*
Sexual domain	37.7 ± 13.8	34.2 ± 12.5	<0.01*	34.0 ± 13.6	<0.01*	34.3 ± 12.3	<0.01*
Hormonal domain	90.8 ± 10.9	90.7 ± 11.2	0.68	92.1 ± 9.6	0.12	93.8 ± 7.9	<0.01*

**Figure 2:**
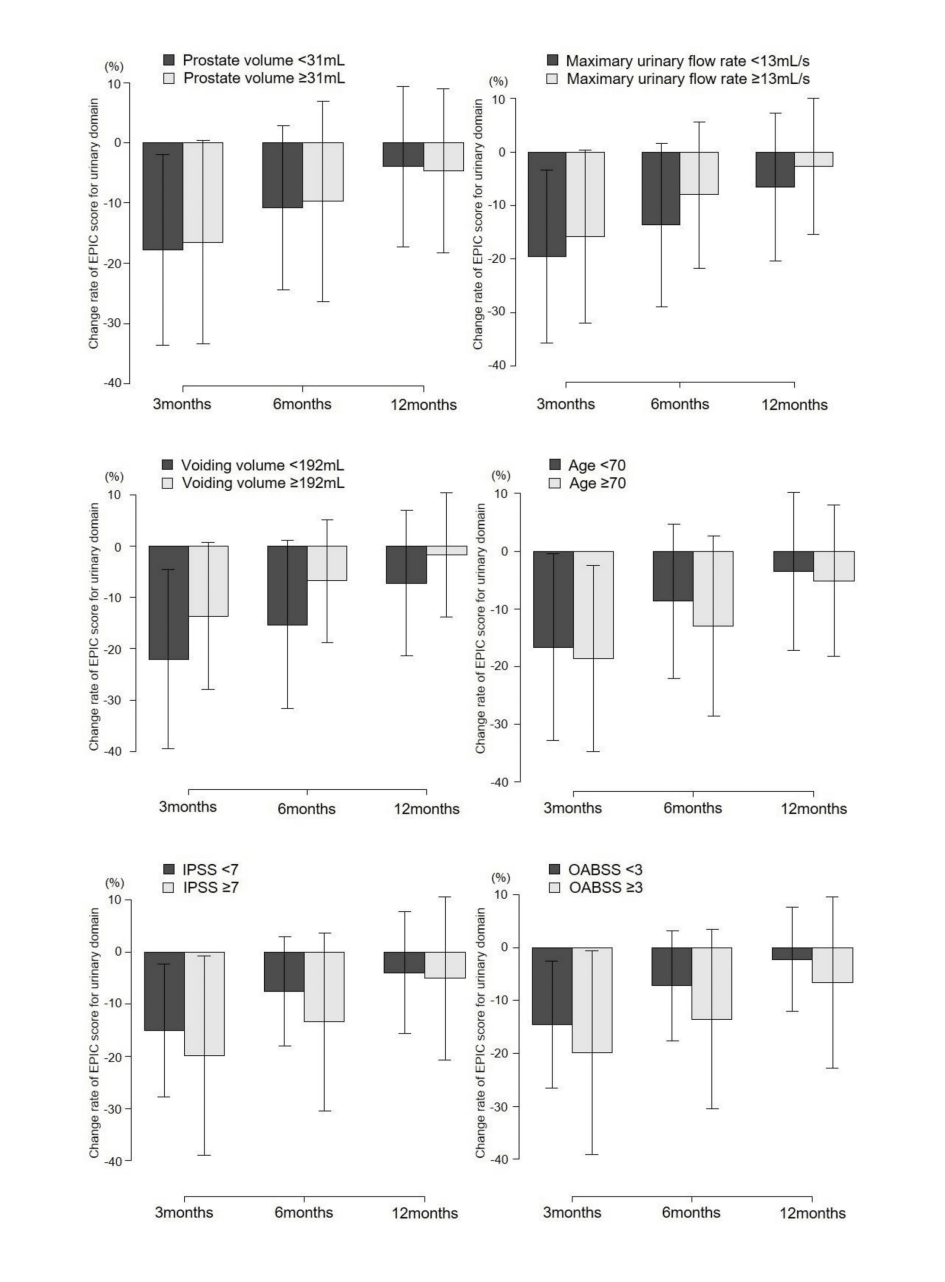
Trends in change rate of the EPIC score for the urinary domain stratified by pretreatment prostate volume, maximum urinary flow rate, voiding volume, age, IPSS, and OABSS EPIC, Expanded Prostate Cancer Index Composite; IPSS, International Prostate Symptom Score; OABSS, Overactive Bladder Symptom Score

Table 3 and Figure 2 compare changes in the EPIC score for the urinary domain at each observation point between groups stratified by pretreatment prostate volume, maximum urinary flow rate, voiding volume, age, IPSS, and OABSS. Patients with a higher OABSS exhibited a significantly greater reduction in scores at three and six months post-treatment, while those with an IPSS of ≥7 showed a significantly greater decline in EPIC scores at six months. No significant differences in score changes were observed based on pretreatment prostate volume, maximum urinary flow rate, or age. However, patients with a pretreatment voiding volume of <192 mL showed significantly lower changes in EPIC scores at 3, 6, and 12 months post-treatment compared with patients who had a voiding volume of ≥192 mL. ROC analysis to determine this cutoff yielded an area under the curve of 0.58 (95% CI: 0.49-0.67).

**Table 3:** Change rate of the EPIC score stratified by prostate volume, maximum urinary flow rate, voiding volume, age, IPSS, and OABSS * indicates statistical significance with p < 0.05. EPIC, Expanded Prostate Cancer Index Composite; IQR, Interquartile Range; IPSS, International Prostate Symptom Score; OABSS, Overactive Bladder Symptom Score

Change rate of EPIC score (%), median (IQR)							
		3 months	p-value	6 months	p-value	12 months	p-value
Prostate volume	<31 mL	-17.0 (-27.5 - -6.3)	0.77	-8.9 (-17.4 - -2.1)	0.68	0.0 (-6.8 - 2.1)	0.69
	≥31 mL	-16.8 (-25.0 - -6.3)		-6.3 (-19.6 - -2.1)		-2.1 (-12.5 - 2.3)	
Maximum urinary flow rate	<13 mL/s	-20.2 (-33.4 - -6.5)	0.11	-12.0 (-20.6 - -2.1)	0.06	-2.4 (-11.7 - 1.6)	0.16
	≥13 mL/s	-13.7 (-24.3 - -4.4)		-5.2 (-15.2 - 0.0)		-0.0 (-5.4 - 2.1)	
Voiding volume	<192 mL	-21.0 (-35.2 - -8.6)	<0.01*	-14.0 (-25.3 - -2.6)	<0.01*	-4.2 (-13.8 - 1.0)	<0.01*
	≥192 mL	-12.7 (-21.9 - -4.2)		-4.6 (-13.4 - -1.1)		0.0 (-4.2 - 2.1)	
Age	<70	-14.8 (-26.1 - -5.2)	0.28	-6.3 (-16.3 - -2.1)	0.17	0.0 (-5.6 - 1.9)	0.30
	≥70	-19.8 (-28.8 - -7.2)		-10.4 (-20.3 - -2.3)		-2.2 (-12.2 - 2.2)	
IPSS	<7	-15.3 (-22.5 - -4.2)	0.06	-4.4 (-13.7 - -2.1)	0.04*	-1.1 (-6.5 - 0.0)	0.82
	≥7	-19.4 (-33.5 - -7.9)		-11.0 (-22.2 - -2.1)		-2.7 (-10.9 - 2.7)	
OABSS	<3	-13.3 (-21.7 - -5.0)	0.05*	-4.4 (-12.7 - -2.1)	0.01*	0.0 (-4.2 - 2.1)	0.10
	≥3	-20.4 (-33.5 - -6.4)		-13.2 (-21.8 - -2.2)		-2.9 (-14.0 - 2.3)	

Table 4 compares patient characteristics between groups with voiding volumes of <192 mL and ≥192 mL. The median age was significantly lower in the higher voiding volume group, and pretreatment IPSS and OABSS scores were also significantly lower in this group. However, no significant differences were observed in other patient characteristics, including the baseline EPIC score for the urinary domain.

**Table 4:** Comparison of patient characteristics between groups with voiding volumes <192 mL and ≥192 mL * indicates statistical significance with p < 0.05. ADT, Androgen Deprivation Therapy; BMI, Body Mass Index; ECOG-PS, Eastern Cooperative Oncology Group Performance Status; EBRT, External Beam Radiation Therapy; EPIC, Expanded Prostate Cancer Index Composite; IQR, Interquartile Range; IPSS, International Prostate Symptom Score; OABSS, Overactive Bladder Symptom Score; NCCN, National Comprehensive Cancer Network

Variable	Voiding volume <192 (n = 84)	Voiding volume ≥192 (n = 104)	p-value
Age (yr), median (IQR)	72 (66-75)	68 (63-70)	<0.01*
ECOG-PS, median (IQR)	1 (1-1)	1 (0-1)	0.08
BMI, median (IQR)	22.9 (20.8-24.9)	24.0 (21.7-25.6)	0.05
NCCN risk classification, n (%)			0.47
Low	36 (42.9)	51 (49.0)	
Intermediate	44 (52.4)	51 (49.0)	
High	4 (4.8)	2 (1.9)	
Prostate volume (mL), median (IQR)	27 (23-31)	27 (23-32)	0.29
Neoadjuvant ADT, n (%)	39 (46.4)	41 (39.4)	0.38
EBRT combination, n (%)	9 (10.1)	4 (4.1)	0.15
Baseline IPSS, median (IQR)	9 (5-14)	6 (4-11)	0.02*
Baseline OABSS, median (IQR)	4 (2-5)	2 (1-3)	<0.01*
Baseline EPIC score (urinary domain), median (IQR)	92.7 (85.3-97.9)	93.8 (87.5-100)	0.09

## Discussion

This study evaluated changes in QOL scores before and after brachytherapy for localized prostate cancer, with a focus on the relationship between urinary-related QOL scores and preoperative findings, including objective urination measurements. The results indicated that QOL, as measured by the urination-related EPIC score, significantly declined at three months post-treatment, before gradually returning to baseline levels over time. However, when patients were stratified by voiding volume assessed through uroflowmetry, those with lower voiding volumes showed a significantly greater decline in urinary function scores at 3, 6, and 12 months post-treatment than did those with higher voiding volumes. Notably, significant differences were observed at all observation points in this classification, suggesting that voiding volume could serve as a predictor of prolonged QOL decline. These findings highlight the potential importance of pretreatment voiding volume in predicting post-brachytherapy QOL outcomes, emphasizing the need to consider this factor carefully when selecting treatment options for localized prostate cancer.

In the early period following brachytherapy, many patients experience urinary dysfunction; however, severe cases are rare, and gradual improvement is typically observed over time [2,11]. Consistent with the findings of this study, previous research has also shown that urinary-related QOL scores decline shortly after treatment but gradually recover to baseline levels [12]. Additionally, pretreatment urinary function has been identified as a predictive factor influencing changes in QOL scores. For instance, a prior study that categorized patients based on their pretreatment IPSS showed that those with a higher IPSS experienced significantly greater declines in QOL scores in the early post-treatment phase, although both groups eventually returned to baseline without prolonged delays [6]. In the present study, comparisons were also made using the IPSS and OABSS, but their impact on QOL score changes was limited. Given that the IPSS and OABSS are symptom-based, their ability to objectively assess patients’ urinary conditions remains a concern.

Therefore, this study evaluated the relationship between urination-related QOL scores and objective pretreatment urination findings, including voiding volume, prostate volume, and maximum urinary flow rate. In our cohort, a cutoff value of 192 mL was established using ROC analysis, and patients were divided into two groups to compare changes in QOL. We selected recovery to baseline EPIC score at 12 months as the endpoint because previous studies have reported that urinary QOL generally returns to baseline within approximately 12 months after brachytherapy [6]. The results demonstrated that pretreatment voiding volume significantly influenced the rate of change in QOL scores from post-treatment to 12 months. When comparing patient backgrounds between these two groups, significant differences were observed in age, OABSS, and IPSS. However, further analysis revealed that these factors had only a limited influence on QOL score changes after treatment. To our knowledge, this is the first study to compare QOL changes using EPIC scores stratified by voiding volume. Accordingly, the cutoff identified in this study was determined exploratorily using ROC analysis. Nevertheless, this finding is important and warrants further investigation in other patient populations. It is possible that a reduction in voiding volume reflects storage dysfunction or voiding dysfunction. In such patients, radiation-induced exacerbation of bladder irritative symptoms may contribute to further deterioration of urinary QOL and delay its recovery. In contrast, the associations between prostate volume and maximum urinary flow rate with QOL scores were limited in this study. Regarding prostate volume, the relatively narrow range of prostate volumes among patients eligible for brachytherapy likely contributed to these findings. Additionally, the results may have been influenced by the fact that 40% of patients received hormone therapy. Previous studies have shown that prostate volume reduction due to hormone therapy does not necessarily improve urinary function [13], and, similarly, no association between prostate volume and post-treatment QOL was observed in this study. Regarding the maximum urinary flow rate, the routine administration of alpha-1 blockers after treatment in nearly all cases likely mitigated urinary dysfunction, thereby minimizing the impact of the pretreatment maximum urinary flow rate on QOL changes [14].

Surgery and radiation therapy are the primary treatment options for localized prostate cancer. Both offer favorable long-term prognoses [15,16], making post-treatment QOL a crucial factor in treatment decisions. Several studies have revealed differences in post-treatment QOL based on treatment methods. Nakai et al. compared QOL scores between patients undergoing robotic-assisted radical prostatectomy (RARP) and those receiving brachytherapy [17], finding that the rate of decline in the urinary function domain of the EPIC score was significantly greater in the RARP group. Specifically, the urinary incontinence score declined more in the RARP group, while the urinary irritative/obstructive score declined more in the brachytherapy group. In patients with pretreatment lower urinary tract symptoms, radical prostatectomy has been shown to improve urinary symptoms [18,19]. This finding suggests that, for patients with benign prostatic hyperplasia or high IPSS scores, surgical treatment may have a lower impact than brachytherapy on urination-related QOL. Meanwhile, a comparative study of post-treatment QOL between intensity-modulated radiation therapy and brachytherapy revealed that urinary domain outcomes were better in the intensity-modulated radiation therapy group, whereas bowel domain outcomes favored the brachytherapy group [20]. Additionally, when comparing QOL between brachytherapy combined with EBRT and brachytherapy alone, the combination therapy resulted in greater impairments in post-treatment QOL across the urinary, bowel, and sexual domains [21,22]. Because the impact of treatment choices on QOL varies by domain, having a pretreatment understanding of the risk factors for post-treatment QOL changes is essential for selecting the most appropriate approach for each patient. Furthermore, obtaining informed consent from patients regarding potential QOL changes is crucial in ensuring that they are fully aware of the risks associated with their treatment options.

Our study had several limitations, including its retrospective design and non-randomized cohort. Additionally, because of the lack of long-term follow-up on QOL changes, the association between pretreatment objective urination findings and long-term post-treatment QOL remains unclear. Furthermore, we did not assess the relationship between post-treatment QOL changes and post-treatment voiding function, and it is possible that post-treatment voiding function may not directly correlate with post-treatment QOL [6]. Despite these limitations, our findings contribute valuable evidence supporting the impact of urinary function on QOL in patients with localized prostate cancer treated with brachytherapy.

## Conclusions

Our findings suggest that pretreatment voiding volume may be associated with subsequent changes in urination-related QOL after brachytherapy for localized prostate cancer. This parameter could potentially serve as a predictor of QOL outcomes and may assist physicians in counseling patients prior to treatment. Nevertheless, the retrospective design and single-institution setting of this study limit the generalizability of our findings. Prospective studies with larger cohorts are warranted to validate these results and clarify their clinical utility.
